# Unexpected profile of sphingolipid contents in blood and bone marrow plasma collected from patients diagnosed with acute myeloid leukemia

**DOI:** 10.1186/s12944-017-0624-1

**Published:** 2017-12-08

**Authors:** Marzena Wątek, Bonita Durnaś, Tomasz Wollny, Marcin Pasiarski, Stanisław Góźdź, Michał Marzec, Anna Chabowska, Przemysław Wolak, Małgorzata Żendzian-Piotrowska, Robert Bucki

**Affiliations:** 1 Department of Hematology, Holy Cross Oncology Center of Kielce, Artwińskiego 3, 25-734 Kielce, Poland; 2 Faculty of Medicine and Health Sciences of the Jan Kochanowski University in Kielce, Kielce, Poland; 3 Department of Pathology and Laboratory Medicine, University of Pennsylvania, Philadelphia, PA USA; 4 Regional Blood Transfusion Center in Bialystok, 15-950 Bialystok, Poland; 5 Department of Hygiene, Epidemiology and Ergonomics Department Medical University of Bialystok, 15-222 Bialystok, Poland; 6 Department of Microbiological and Nanobiomedical Engineering, Medical University of Bialystok, 15-222 Bialystok, Poland

**Keywords:** Sphingolipids, Ceramide, S1P, Acute myeloid leukemia, Hematological malignancies, Plasma gelsolin

## Abstract

**Background:**

Impaired apoptotic pathways in leukemic cells enable them to grow in an uncontrolled way. Moreover, aberrations in the apoptotic pathways are the main factor of leukemic cells drug resistance.

**Methods:**

To assess the presence of potential abnormalities that might promote dysfunction of leukemic cells growth, HPLC system was used to determine sphingosine (SFO), sphinganine (SFA), sphingosine-1-phosphate (S1P) and ceramide (CER) concentration in the blood collected from patients diagnose with acute myeloblastic leukemia (AML; *n* = 49) and compare to values of control (healthily) group (*n* = 51). Additionally, in AML group concentration of SFO, SFA, S1P and CER was determined in bone marrow plasma and compared to respective values in blood plasma. The concentration of S1P and CER binding protein – plasma gelsolin (GSN) was also assessed in collected samples using immunoblotting assay.

**Results:**

We observed that in AML patients the average SFO, SFA and CER concentration in blood plasma was significantly higher (*p* < 0.001) compare to control group, when blood plasma S1P concentration was significantly lower (*p* < 0.001). At the same time the CER/S1P ratio in AML patient (44.5 ± 19.4) was about 54% higher compare to control group (20.9 ± 13.1). Interestingly the average concentration of S1P in blood plasma (196 ± 13 pmol/ml) was higher compare to its concentration in plasma collected from bone marrow (154 ± 21 pmol/ml).

**Conclusions:**

We hypothesize that changes in profile of sphingolipids concentration and some of their binding protein partners such as GSN in extracellular environment of blood and bone marrow cells in leukemic patients can be targeted to develop new AML treatment method(s).

## Background

Glycosphingolipids (GSLs) are a family of biomolecules characterized by their pleiotropic effects on cell function including signalling, regulation of cell proliferation and apoptosis. Certain family members act as bioactive lipids, relaying outside-in signals during inflammatory reactions. The complexity of GSL biological functions is derived in part from their localization to both intra- and extracellular compartments. A number of GSLs are involved in the differentiation of acute myeloid leukaemia (AML) cells, as indicated by their increased expression in the bone marrow of patients with AML compared to healthy subjects [1]. Within the GSL family, sphingosine (SFO) functions as an endogenous mediator of the apoptotic signal. When HL-60 cells were exposed to SFO or its methylated derivative, N,N-dimethylsphingosine, intranuclear DNA fragmentation and morphological changes characteristic to apoptosis were observed [2]. Certain proteins such as gelsolin (GSN) may act as universal carriers/scavengers of sphingosine-1-phosphate (S1P), serving to interfere with S1P activity [3–5]. Such interactions may be of importance in settings where the concentration of both substances is outside of homeostatic ranges [6]. GSN changes might be of grate importance since it is a multifunctional Ca^2+^/polyphosphoinositide-regulated actin-binding protein [7, 8]. In human blood plasma, the GSN concentration is 150–300 μg/ml, and muscle is its principal birthplace [9, 10]. Based on gelsolin’s ability to depolymerize actin filaments, its function as actin scavenger has been emphasized [11]. Another discussed role of gelsolin is its involvement in regulation of inflammatory and cancer-promoting processes through interactions with different bioactive lipids including LPA, PAF and LPS, S1P [12–15]. Human blood plasma gelsolin levels decline markedly in a variety of acute clinical conditions including major trauma [16], prolonged hyperoxia [17], acute oxidant lung injury [18] malaria [19], sepsis [15] and liver injury [20, 21]. Interestingly, the level of GSN is significantly increased in HIV-1 infected patients compared to healthy volunteers [22]. The connection between decreasing gelsolin amount and tissue injury and the possibility of therapeutic top up of GSN to stop biological pathways of damaging cascades are active areas of investigation [23].

The over-expression of sphingosine kinase-1 in chemosensitive HL-60 cells results in a significant inhibition of apoptosis, which is mediated by inhibition of mitochondrial cytochrome c. The incubation of chemo-resistant cells with cell-permeable ceramide (CER) leads to the inhibition of sphingosine kinase-1 and supresses apoptosis. Moreover, F-12509a, a new inhibitor of sphingosine kinase induces CER accumulation and reduced S1P, which initiates the same sensitivity to chemotherapy as chemo-sensitive and chemo-resistant cells; This effect is inhibited by adding S1P or overexpressing sphingosine kinase-1 [24]. Inhibition of the sphingosine kinase-1 activity coupled with an increase in CER generation has been shown in chemosensitive HL-60 cells. On the contrary, the high activity of sphingosine kinase-1 but no ceramide generation during anti-cancer treatment has been shown in chemo-resistant HL-60 cells. S1P is present in plasma with high-density lipoproteins and albumin complexes [25]. There has been a significant effort to assess their involvement in cell proliferation, differentiation and apoptosis during malignant development. It is well documented that the increased intracellular availability of CER induces DNA degradation and death of human leukemic HL-60 and U937 cell lines [26]. Using traditional in vitro cellular models (HL-60 cell line), many early studies revealed that changes in sphingolipid metabolism in leukemic cells can induce apoptosis and differentiation, which may provide therapeutic benefit [27]. The increase in ceramide concentration correlates with the differentiation of HL-60 cells induced by treatment with vitamin D3 that is associated with increased of neutral SMase activity in cell extracts and ceramide relesaed via hydrolysis of sphingomyelin [27]. CER accumulation plays also a key role in the death of AML-M2 cells induced by FTY720P [28] and blockage of protein kinase C increases the pro-apoptotic influence that ceramide has on human leukemia cells [29]. Manipulation of CER metabolism to increase its production and accumulation synergistically enhances the apoptotic effect of resveratrol in HL60 cells. More importantly, gene expression analysis showed that resveratrol induces apoptosis by over-expression of genes that generate CER and a decrease in the expression of sphingosine kinase-1 and genes that synthesize glukozylceramid [30]. The accumulation of ceremide during differentiation was also observed in EL4 thymoma cells, cerebral Purkinji cells, and U937 monoblastic leukemia cells [27]. Furthermore, in lymphoblastic leukemia cells apoptosis in response to doxorubicin and vinca alkaloid is promoted by the accumulation of ceramide [31]. Clinical observations indicate that anti-leukemic agents, effective in eradicating blasts, are relatively ineffective in eliminating leukemic progenitor cells as indicated by a high recurrence rate in cases of acute myelogenous leukaemia with high index of complete remission (70%). This interpretation underscores the natural chemo-resistance of cells that form the myeloid leukaemia progenitor compartment. In the last few years, studies have shown that similar cell perturbations may cause effects such as quick apoptotic death, differed mitotic death or non-lasting cytostatic effect. Intracellular signalling is responsible for the specific response to a given stimuli. These signals are mediated in part by ceramide (cell death signal mediator) and diacylglycerol and phosphoinositide-3 phosphates (PI3P) (survival signal mediator). These observations underline different possibilities of pharmacological manipulations favouring the death of cells or their resistance to anti-cancer agents [32].

## Methods

### Specimen collection

Human blood and bone marrow specimen collection was performed in the Holy Cross Oncology Center of Kielce. The experiments were performed according to the principles outlined in the Declaration of Helsinki and approved by the Ethical Committee of the Jan Kochanowski University in Kielce. At the time of patient recruitment, written consent was obtained from all subjects and all patients gave their informed consent prior to inclusion in the study. We collected blood and bone marrow plasma from patients with AML and blood samples from healthy subjects. Samples were obtained from patients while undergoing required diagnostic testing. Control samples were obtained from healthy volunteers during testing to exclude hematological diseases. Informed consent was obtained prior to collection and use of the test material. The samples of marrow and whole blood were placed into EDTA tubes (K2 EDTA 5.4 mg) at room temperature, and were centrifuged at 3200 rpm for 10 min to separate plasma which was then transferred to fresh plastic tubes and frozen −70 °C until use. Clinical and laboratory characteristics of the patient groups are given in Table 1. AML was diagnosed in patients using standard hematological identification. Diagnosis of AML was confirmed by phenotypic and cytological bone marrow testing. Because of the small sample size we did not analyze sphingolipid concentrations relative to cytogenetic group or FAB diagnosis.

**Table 1:** Clinical characteristics of AML patients and a reference group

AML Patients (*n* = 49)			
FAB Subtype	Number of patients	Age	Sex F/M
AML M0	2	53 ± 0	2/0
AML M1	6	57.5 ± 19.25	5/1
AML M2	15	58.8 ± 15.53	8/7
AML M4	8	70.25 ± 12.89	5/3
AML M5	4	63 ± 20.92	2/2
AML M5b	5	62.8 ± 9.4	2/3
AML/MDS	9	59.44 ± 11.51	4/5
All patients	49	61.14 ± 15.07	28/21
Reference group (*n* = 51)			
Control group	51	58.68 ± 19.77	24/27

### Evaluation of S1P in blood plasma and CSF samples

The sphingosine-1-phosphate concentration was measured by the method described in [33]. Briefly, acidified methanol and an internal standard (30 pmol of C_17_-S1P, Avanti Polar Lipids) were added to 250 μl of plasma or CSF and then the samples were ultrasonicated in ice-cold water for 1 min. The lipids were then extracted with chloroform, 1 M NaCl, and 3 N NaOH. The alkaline aqueous phase containing S1P was transferred to a fresh tube. Residual S1P in the chloroform phase was re-extracted twice with a methanol/1 M NaCl (1:1, *v*/v) solution and then all aqueous fractions were combined. The amount of S1P was determined indirectly after dephosphorylation to sphingosine using alkaline phosphatase (bovine intestinal mucosa, Fluka, Milwaukee, WA). To improve the extraction yield of released sphingosine, chloroform was carefully placed at the bottom of the reaction tubes. The chloroform fraction containing dephosphorylated sphingoid base was washed three times with alkaline water (pH adjusted to 10.0 with ammonium hydroxide) and then evaporated under a nitrogen stream. The dried lipid residues were re-dissolved in ethanol, converted to their o-phthalaldehyde derivatives, and analyzed using an HPLC system (ProStar, Varian Inc.) equipped with a fluorescence detector and a C18 reversed-phase column (Varian Inc. OmniSpher 5, 4.6150 mm). The isocratic eluent composition of acetonitrile (Merck): water (9:1, *v*/v) and a flow rate of 1 ml/min were used. The column temperature was maintained at 33 °C by use of a column oven.

### Immunoblotting analysis

After being thawed, gel sample buffer was added to plasma or bon marrow samples that were then boiled and subjected to electrophoresis on 10% polyacrylamide gels in the presence of SDS. Recombinant human plasma gelsolin (rhpGSN) was loaded as a standard in each gel in a concentration range comparable to the gelsolin concentration in the samples. After electrophoresis, proteins were transferred to PVDF membranes (Amersham, Biosciences Little Chalfont, UK), which were blocked by incubation in 5% (*w*/*v*) non-fat dry milk dissolved in TBS-T (150 mM NaCl, 50 mM TRIS, 0.05% Tween 20, pH = 7.4). Following transfer, proteins were probed with a monoclonal anti-human gelsolin antibody (Sigma, St Louis, MO, USA). Both antibodies were used at 1:10,000 dilution in TBS-T. HRP-conjugated secondary antibodies were used at 1:20,000 dilution in TBS-T. Immunoblots were developed with the Fuji Film LAS-300 system using an ECL Plus HRP-targeted chemiluminescent substrate (Amersham, Biosciences Little Chalfont, UK). Densitometry analysis was performed using Image Gauge (version 4.22) software (Fuji Photo Film Co, USA).

### Statistical analysis

Data are reported as a mean ± SD. Differences between means were evaluated using the Student’s t-test, with *p* < 0.05 being taken as the level of significance.

## Results

### Outline of blood sphingolipids

**Fig. 1:**
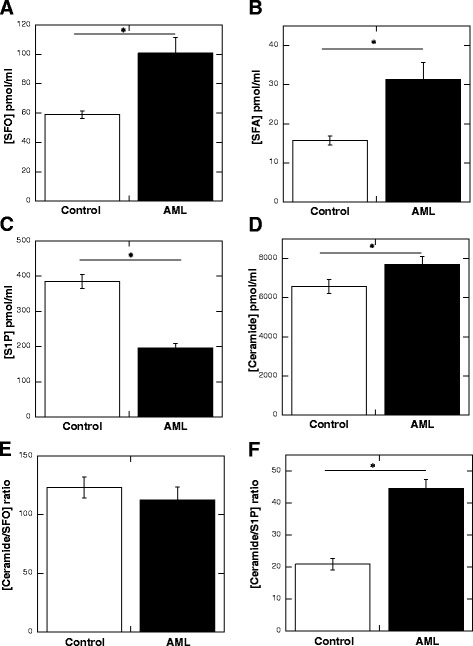
Concentration of SFO (panel **a**), SFA (panel **b**), S1P (panel **c**) and ceramide (panel **d**) in blood plasma samples collected from subject included in control group (*n* = 51) and AML patients (*n* = 49). The ratio of ceramide to SFO and ceramide to S1P in control and AML blood plasma samples are shown on panel **e** and **f**, respectively

**Fig. 2:**
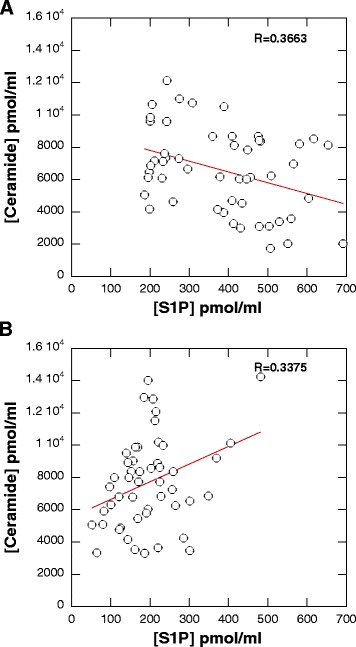
Correlation plot of ceramide versus S1P concentration in blood plasma collected from subjects included in the control group (panel **a**) and AML patients (panel **b**). R’ values were calculated using linear curve fit

We observed a statistically significant increase in the concentration of SFO, SFA (*p* < 0.001) and CER (*p* < 0.05) in the blood of patients with AML compared to the control group (Fig. 1a–d). Conversely, the S1P concentration in blood plasma from AML patients was lower (*p* < 0.001) compared to the control group (Fig. 1c). Based on the assumption that the ultimate cellular effect of molecules with opposite functions (CER promotes apoptosis while S1P promotes proliferation) is based on their relative abundance we evaluated the ratio of ceramide to SFO and ceramide to S1P in both control and AML blood plasma samples (Fig. 1e). The ratio between CER/S1P in the blood of AML patients was significantly higher compared to the control group (Fig. 1f, *p*<0.001). Additionally, we assessed the correlation between concentrations of CER and S1P in the blood plasma of the AML patients and control group (Fig. 2a, b). Interestingly, a weak positive correlation was been found between the concentration of ceramide and S1P in the blood of AML group (*R* = 0.3375). In the control group this trend was opposite (*R* = 0.3663) (Fig. 2a and b, respectively).

### Characterization of bone marrow sphingolipids

**Fig. 3:**
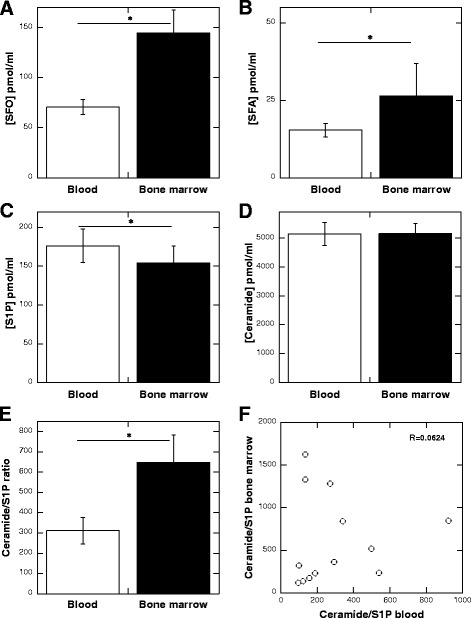
Concentration of SFO (panel **a**), SFA (panel **b**), S1P (panel **c**) and ceramide (panel **d**) in blood and bone marrow collected from patient diagnosed with AML (*n* = 13). Values of ceramide to S1P ratio in blood and bone marrow shown in panel (**e**). Correlation plot of values of ceramide to S1P ratio in blood to values of ceramide to S1P ratio in bone marrow represents panel (**f**)

**Fig. 4:**
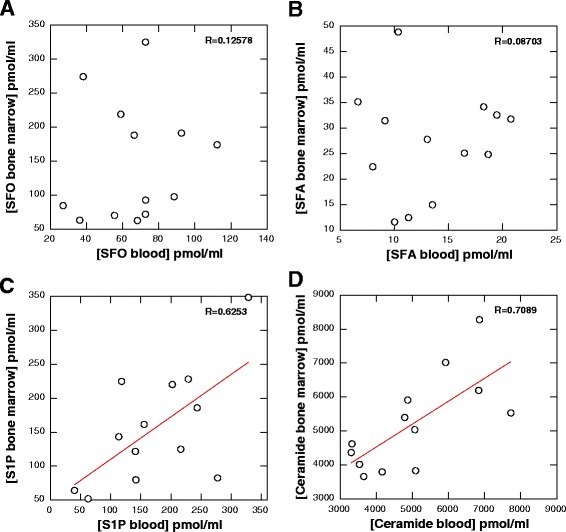
Correlation plot between values of SFO (panel **a**), SFA (panel **b**), S1P (panel **c**) and ceramide (panel **d**) concentration in blood plasma versus their concentration in analogous samples of bone marrow plasma. All specimens were collected from patients diagnosed with AML (*n* = 13). R’ values were calculated using linear curve fit

Although 49 AML patients were included in the study, only 13 bone marrow samples were collected at volumes large enough to perform HPLC. Despite these limitations, comparison of SFA, SFO, S1P and CER blood and bone marrow plasma concentrations revealed measurable differences. The concentrations of SFO and SFA were significantly higher (*p* < 0.001) in bone marrow plasma (Fig. 3a and b), while the concentration of S1P was significantly higher (*p* < 0.01) in blood plasma (Fig. 3c). There was no difference between the concentration of ceramide in bone marrow and blood plasma collected from AML subjects (Fig. 3d). The ratio between ceramides and S1P in the bone marrow was significantly higher (*p* < 0.001) than in the blood from AML patients (Fig. 3e). The correlation of the ratio of the CER/S1P in the blood as compared to the bone marrow did not reveal any trend (*R* = 0.0624). The ceramide/S1P ratio in the bone marrow and blood from AML patients had a low positive correlation (R = 0.0624, Fig. 3f). The correlation values between the concentration of sphingolipids (SFO, S1P, ceramide) in the blood and bone marrow of patients with AML was positive (Fig. 4a–d). No trend was observed when the correlation of SFA in bone marrow versus SFA in blood plasma was plotted. Interestingly a strong positive correlation between S1P (*R* = 0.6253) and ceramide (*R* = 0.7089) concentration in bone marrow and blood plasma (Figs. 4c, d) was recorded.

### Gelsolin concentration in blood and bone marrow plasma

**Fig. 5:**
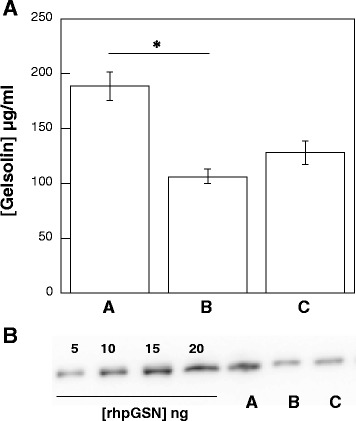
Plasma gelsolin concentration in blood plasma of control subjects (*n* = 51), ALM patients (*n* = 49) and bone marrow plasma collected from patients diagnosed with ALM (*n* = 13) (panel **a**) (each sample characterized individually). Western-blot analysis of combined plasma (A and B) or combined bone marrow plasma (C) in comparison to standard of recombinant human plasma gelsolin (rhpGSN); for combined samples a pool of plasma was made by mixing 10 μl of plasma collected from each individual subject (Panel **b**)

As shown in Fig. 5, in a limited number (*n* = 13) of bone marrow samples, the range of measured gelsolin concentration was 76.5–158.9 μg/ml and was comparable to lower plasma gelsolin concentration in plasma of ALM patients (45.3–132.7 μg/ml) when determined using quantitative immunoblotting. The average gelsolin concentration in plasma obtained from subjects included in control group was within the range of 87.9 ± 274.3 μg/ml. Comparing all samples, the lowest gelsolin concentrations were observed in blood of patients diagnosed with ALM.

## Discussion

There is increasing evidence to support the hypothesis that the use of infrared and many chemotherapeutic agents on leukemic cells results in apoptosis in part through changes in sphingolipid metabolism. It has been shown that the accumulation of ceramide produced in the process of sphingomyelin hydrolysis as well as in the de novo synthesis play an important role as a mediator of the leukemic cell death [34]. The CD95(APO-1/Fas) antigen plays an important role in activating apoptosis in leukemic T cell acute lymphoblastic leukaemia as well as mediating cellular DNA fragmentation. Interestingly, previous studies demonstrate that ceramide plays an important role in the up regulation of CD95(APO-1/Fas) [31]. Additionally, doxorubicin, daunorubicin, and nucleoside analogues such as cytarabine or fludarabine, which are used in induction chemotherapy of acute leukemias, are able to induce apoptosis both through their incorporation into cellular DNA as well as by stimulating ceramide generation. Finally, several reports suggest that the cytostatic drugs AraC, fludarabin, and VCR activate apoptosis by inducing ceramide production [34].

In chronic myeloid leukemia S1P enhances the anti-apoptotic protein Mcl-1 (myeloid cell leukemia-1) and its binding to the S1P receptor type 2 (S1PR2) [35]. In acute myeloid leukemia, S1P induces mitogenic signaling through activation of NF-kB [28] resulting in inhibition of apoptosis in U937 and HL-60 cells [29]. Furthermore, S1P inhibits classical apoptosis in T acute lymphoblastic leukemia (T-ALL) [28] and SPHK1 level is increased in B-ALL, contributing to the development of murine BCR/ABL1 ALL [19]. Results from these studies and others lead to the conclusion that decreased levels of ceramide and/or S1P are important regulators for the resistance of leukemic cells to drug-induced apoptosis [36, 37]. This motion is also supported by reported correlation of SK1 and S1P receptor expression in tumours with patient survival and tamoxifen resistance in ER+ breast cancer [38, 39]. On the other hand in cancer associated with inflammation in contrast to oncogenic SphK1, either overexpression or down-regulation of SphK2 has been shown to inhibit cell growth and promote apoptosis in a cell-dependent manner [40].

The studies shown here demonstrate that dynamic changes in the extracellular concentration of lipids belonging to sphingolipid signal transduction have practical significance for AML patients. They suggest that, as in tumours derived from epithelial cells, the regulation of S1P and ceramide-mediated signal transmission may be a target for the treatment hematopoietic malignancies. Indirect evidence for this motion is the study demonstrating that de novo ceramide production induces spontaneous neutrophil apoptosis through the activation of the caspase pathway. Studies on the role of ceramide performed using the Molt-4 human leukaemia cell line have confirmed that the ceramide molecules synthesized de novo activate apoptosis in the presence of etoposide [41]. Ceramide induces apoptosis through the mitochondrial pathway, partly through the influence of Bcl-2 proteins. Studies conducted by Bose et al. demonstrate that daunorubicin induces apoptosis of leukaemia U937 and P388 by increasing the levels of ceramide [42]. The abillity of FTY720P to induce apoptosis of AML cells in leukemic mice suggests the ability of extracellular S1P to affect cellular signalling pathway initiated by the interaction of S1P with cell surface receptors [28]. The outcome of sphingolipids concentration changes during ALM development might also be affected by changes of plasma level of their protein binding regulators such as plasma gelsolin. Changes in plasma gelsolin can result from disturbed gelsolin-actin interaction, binding of gelsolin to cellular mediators, or modulation of gelsolin synthesis in response to actin release [3] and might affect the extracellular network of biopolymers [43].

Our experiments demonstrate significantly increased plasma levels of sphingolipids in patients with acute leukaemia, which is associated with decrease of plasma gelsolin. The concentrations of SFO, SFA and ceramide are higher in AML patients while the concentration of S1P is reduced. It is possible that impaired sphingolipid metabolism plays a role in AML disease progression. Previously described studies demonstrating changes in the activity of enzymes involved in sphingolipid metabolism (glucocerebrosidase, galactocerebrosidase, sphingomyelinase, acid phosphatase) in leukemic cells support this hypothesis [19]. These conclusions are further supported by observations demonstrating differences in sphingolipid expression between healthy donors and AML patients (AML patients had higher Lc3 and nLc4 expression than healthy subjects) [1].

The current dogma is that platelets are the main source of S1P in the blood. Low platelet levels in AML patients may partly explain the observation of low plasma concentrations of S1P, particularly in patients who experience, during the so called repression mechanism, the reduction of level of platelets in the blood [44]. Generated data strongly indicate reduced concentrations of lipids of the sphingolipid pathway involved in signal transmission and suggest their participation in the development of AML. The findings also suggest the involvement of sphingolipids in AML cell differentiation. It is known that SFO and ceramide exhibit pro-apoptotic properties, while S1P promotes cell growth and survival [17]. High concentrations of pro-proliferative (S1P) and low concentrations of pro-apoptotic sphingolipids (ceramide) might be expected in patients with a disease with active leukemic cell proliferation. Furthermore, our data suggest that the measured phospholipid concentration profile may be lacking some informational molecule, which initiates cell death. This observation is in line with previous reports indicating that low levels of ceramide and elevated sphingolipid pathway enzymes are associated with acute leukemia cell chemoresistance [34]. Both sphingolipids and sphingolipids pathway enzymes may represent potential therapeutic targets. In various cancers, SPHK1 mRNA was significantly higher compared to normal tissue and the bone marrow from AML patients has much higher SPHK1 gene expression controls [45]. Overall, a descriptive nature of presented data and lack of functional studies that prove the implication of these findings in leukemogenesis represent a limitation of our study that in the future should be address with knockdown experiments, use of inhibitors to manipulate glycosphingolipids levels in vitro or in mouse models of blood cancer development.

## Conclusions

Based on our results, we conclude that sphingolipids and their associated pathwyas may be used for either the identification or forecast for the development of AML. We also predict that the use of sphingolipids analogs will be included in the spectrum of AML treatment.

## Abbreviations

AML: Acute myeloblastic leukemia
CER: Ceramide
FTY720P: phosphoric acid ester of myriocin (structural analog of sphingosine)
HPLC: High performance liquid chromatography
LPA: Lysophosphatidic acid
LPS: Lipopolysaccharides
PAF: Platelet activating factor
S1P: Sphingosine-1-phosphate
SFA: Sphinganine
SFO: Sphingosine
SPHK1: Sphingosine kinase 1
VCR: Vincristine
